# Controversies in the Surgical Management of Spinal Cord Injuries

**DOI:** 10.1155/2012/417834

**Published:** 2012-05-14

**Authors:** Ahmed M. Raslan, Andrew N. Nemecek

**Affiliations:** Department of Neurological Surgery, Oregon Health & Science University, Mail Code CH8N, Portland, OR 97239, USA

## Abstract

Traumatic spinal cord injury (SCI) affects over 200,000 people in the USA and is a major source of morbidity, mortality, and societal cost. Management of SCI includes several components. Acute management includes medical agents and surgical treatment that usually includes either all or a combination of reduction, decompression, and stabilization. Physical therapy and rehabilitation and late onset SCI problems also play a role. A review of the literature in regard to surgical management of SCI patients in the acute setting was undertaken. The controversy surrounding whether reduction is safe, or not, and whether prereduction magnetic resonance (MR) imaging to rule out traumatic disc herniation is essential is discussed. The controversial role of timing of surgical intervention and the choice of surgical approach in acute, incomplete, and acute traumatic SCI patients are reviewed. Surgical treatment is an essential tool in management of SCI patients and the controversy surrounding the timing of surgery remains unresolved. Presurgical reduction is considered safe and essential in the management of SCI with loss of alignment, at least as an initial step in the overall care of a SCI patient. Future prospective collection of outcome data that would suffice as evidence-based is recommended and necessary.

## 1. Introduction

Traumatic spinal cord injury (SCI) affects over 200,000 people in the USA, with nearly 10,000 new injuries reported annually [1–3]. People affected by SCI are usually young (average age 32 years) and life expectancy is marginally lower when compared with a non-SCI population. Hence, the ramifications of the injury itself as well as the medical decisions made can result in enormous economic burden and social cost [4]. Long-term outcome after SCI comprises a combination of the initial extent of the injury, natural recovery from injury, medical interventions, rehabilitation, and social/community reintegration.

Several components play a role in management of SCI. Initially, acute management includes medical agents administered with the goal of reducing secondary injury cascade, and an initial surgical treatment that usually includes either all or a combination of reduction, decompression, and stabilization. Physical therapy and rehabilitation, which can lead to a significant impact on overall recovery is an additional component. Finally, late onset SCI problems, such as bowel and bladder dysfunction, pain, spasticity, and problems with automatic breathing, play a role.

This paper focuses on the surgical management of SCI in the acute setting. Later interventions for other sequelae of SCI, for example, spasticity management, and chronic pain management are not discussed.

Early intervention has been a focus of treatment for enhancing neurological recovery. Research using animal models has provided evidence that early decompressive surgery can lead to improved neurological recovery after SCI [5, 6], which highlights the importance of early intervention. Unfortunately, the number of human spinal surgical studies using prospective, randomized, or controlled methodologies is limited.

A review of the current literature reveals varying results, some indicate the potential neurological benefit associated with “early” spinal decompression [7, 8] while others do not [6, 9–11]. Choice of surgical procedure for individual types and severity of SCI is also controversial. In this paper, we place emphasis on currently available evidence for indications and outcomes of the most commonly available surgical options including presurgical reduction as well the choice of the surgical option for any given particular injury pattern.

## 2. Initial Closed Reduction

Traumatic cervical spine fractures or dislocations are often associated with cervical SCI as a result of several mechanisms, including narrowing of the cervical spinal canal or by direct displacement of one cervical spine component with direct focal compression on the spinal cord. Reduction of the cervical spine displacement can restore the spinal cord diameter or relieve the focal compression on the spinal cord. As reported in a few case reports or small case series [9, 12–14] the argument is that focal traumatic disc herniation, which is often associated with traumatic spine injuries, can lead to worsening neurological condition if reduction is attempted.

We review the controversy surrounding whether reduction is safe, or not, and whether prereduction magnetic resonance (MR) imaging to rule out traumatic disc herniation is essential in the next few paragraphs.

Several authors [9, 10, 12–14] believe that traumatic disc herniation associated with fracture-dislocation or facet dislocation increases the risk of spinal cord herniation after reduction. However, the association of a high percentage of disc herniation with dislocated facets (42%) [10] and the absence of a clear relation between outcome and pre- or postreduction MR imaging results has raised questions about the real value of obtaining MR images before reduction.

Cumulative evidence suggests that early reduction influences outcome [10, 15–18], hence delaying reduction by obtaining MR images, which requires transport of a patient with an unstable spine fracture to the MR imaging suite, may influence outcome and requires adequate justification. Initial closed reduction is generally part of the early intervention paradigm in treating acute SCI and is generally undertaken on an urgent basis. The finer details of the reduction technique are not covered in this paper. However, in summary, the technique is generally performed using Gardner-Wells tongs, Crutchfield, or Halo rings. No data is available to support the preferred use of one instrument over another. Gardener wells and Halo rings models are available that are now compatible with MR imaging.

Close monitoring of vital hemodynamic performance parameters are critical during application of traction. Craniocervical traction typically begins with a small weight that is gradually increased until anatomic alignment or near-alignment is achieved. No specific recommendations regarding ideal or maximum weight are advised. However, approximately 5 pounds per level is generally recommended after 10 pounds for the head are added. Caution and clinical judgment are required during traction with careful consideration paid to whether the patient is awake or not, paralyzed or not, and X-ray control to avoid overdistraction. In awake patients, the reduction attempt end point is determined when reduction and realignment, or worsening pain or neurological function is achieved. In comatose patients, clinical judgment or X-ray evidence determines the reduction attempt end point rather than overdistraction. If reduction is deemed impossible, further imaging is required to determine the cause of irreducibility, which is usually anatomical and usually requires an open surgical reduction [19, 20].

In an attempt to develop SCI management guidelines, the cervical spine trauma group developed jointly by the American Association of Neurological Surgeons (AANS) and Congress of Neurological Surgeons (CNS), has reviewed all pertinent literature regarding C spine reduction through the year 2001. Their resultant cumulative meta-analysis found 1200 cases of closed reduction with class III evidence. Overall permanent neurological injury rate was <1% (the equivalent of 11 patients). In those 11 patients, two had root injuries, two had ascending spinal cord injuries at the time of reduction, and seven had a worse American Spinal Injury Association (ASIA score), but neither the nature nor the cause of this change in ASIA score was identified. Twenty other patients had temporary neurological deterioration, the causes of which were: over distraction, disc herniation, unrecognized rostral injury, epidural hematoma, or spinal cord edema [21].

The review group's final conclusion was that closed reduction of fracture-dislocation injuries of the cervical spine by traction-reduction was safe and effective for the reduction of spinal deformity in awake patients. The group concluded that with closed reduction approximately 80% of patients will experience injury reduction. Overall permanent neurological complication rate for closed reduction was approximately 1% and the associated risk of a transient injury was 2% to 4%.

Closed traction-reduction would seem to be safer than manipulation under anesthesia. There are numerous causes of neurological deterioration in patients with unstable cervical spine injuries. These include inadequate immobilization, unrecognized rostral injuries, overdistraction, loss of reduction, cardiac, respiratory, and hemodynamic instability. Although prereduction MR imaging demonstrates disc herniation in up to half of patients with facet subluxation injuries, the clinical importance of these findings is questionable. The use of prereduction MR imaging has not been shown to improve the safety or efficacy of closed traction reduction in awake patients. MR imaging before fracture-dislocation reduction may result in unnecessary delays in accomplishing fracture realignment and decompression of the spinal cord. Class III evidence exists in support of early closed reduction of cervical fracture-dislocation injuries with respect to neurological function, therefore prereduction MR imaging in this setting is not necessary. The ideal timing of reduction is unknown, but many investigators favor performing reduction as soon as is possible after injury to maximize the potential for neurological recovery. In those patients where attempted closed reduction of a cervical fracture injury fails a higher incidence of anatomic obstacles to reduction, including facet fractures and disc herniation is observed. In patients where closed reduction fails further detailed radiographic studies should be undertaken before attempts at open reduction. Patients with cervical fracture-dislocation, who cannot be examined, because of head injury or intoxication, cannot be assessed for neurological deterioration during attempted closed traction-reduction. For this reason, an MR imaging study before an attempted reduction is recommended as a treatment option [21]. In patients with bilateral locked facets resulting in closed reduction failure, surgical reduction in incomplete tetraplegic patients is supported by class II evidence [22–24].

## 3. Timing of Surgery

The role that timing of surgical intervention plays in SCI management remains one of the most controversial topics. Despite immense research efforts related to SCI treatment, neurological recovery and overall outcome remains poor. Any treatment components that can contribute to improved outcomes are welcomed, and early surgical intervention still offers hope.

An increasing number of studies supporting the existence of so-called “secondary injury” in cervical spine trauma have sparked interest in the concept of “time-window.” Secondary injury is defined a cascade of events initiated by trauma and involves vascular changes, electrolytes shifts, excitotoxic neurotransmitters accumulation, inflammation, and loss of energy metabolism [25, 26]. The first clinical human trial to address and highlight the concept of secondary injury was the National Acute Spinal Cord Injury Study (NASCIS) trial. In the NASCIS-2 trial, methyl-prednisolone was used to treat acute SCI within a specific window of time, and although results were statistically marginally significant, some clinical evidence to support the secondary injury concept was indicated [27]. The fundamental question was whether early surgery would lead to improved outcome or not, if not performed within what was a poorly defined “time-window.” Before discussing surgical timing any further, we would like to highlight the heterogeneity of human SCI, for example, population demographics, mechanism of injury, injury pathology, injury severity and the level of injury. This heterogeneity makes it difficult to conclude whether early surgery for a SCI is helpful or not. It is possible that certain SCI patient subsets may benefit more than others while some may not benefit at all. Animal data strongly suggests that early decompression my result in improved outcome after SCI. Many investigators have examined different injury models in different animals for varying periods of time, the vast majority of which have shown some beneficial effect from early decompression [5, 28–38]. However, this experimental evidence of benefit was not translated to clinical evidence with the same degree of certainty. This was most likely due to patient heterogeneity and the inherent difficulty in study design. In an attempt to determine whether early surgery improves outcome or not, some investigators attempted a meta-analysis of related and current SCI literature to form an evidence based answer to the question [39, 40]. A Toronto group of investigators reviewed data that included all human SCI trials conducted between 1966 and 1998 and concluded that despite strong experimental evidence there was no clear census as to the appropriate timing of surgical intervention and that there was no compelling evidence that early surgical decompression influences patient neurological outcome [22, 23, 39]. The basis of their conclusion was that most studies where only class III. On the other hand, an Italian group of investigators reviewed data that included human SCI surgical trials undertaken between 1996 and 2000. They concluded that despite the presence of some statistically significant neurological advantage with early surgery in an incomplete injury subset of patients, they were unable to determine with any confidence a neurological advantage to surgery within 24 hours of injury in any of the groups they studied [40]. The Toronto group recently updated their meta-analysis data and included studies between 2000 and 2005. There were no changes in the overall recommendations from their earlier meta-analysis [23]. Interestingly, in a multicenter retrospective study that included 36 North American centers information regarding the timing of intervention in acute SCI varied widely. Surgery was performed <24 hours after injury in 23.5% of patients, between 25 and 48 hours after injury in 15.8% of patients, between 48 and 96 hours in 19% of patients, and more than 5 days post injury in 41.7% of patients [41]. The results of this study clearly revealed a lack of consensus on optimum timing of surgical treatment. A recent retrospective study, that looked at the outcome of early surgical management versus late or no surgical management after acute SCI in all acute nonpenetrating, traumatic SCI from 1995 to 2000 found no difference in neurological or functional improvements between the early versus the late surgical group [42].

The only class I evidence study addressing this issue is by Vaccaro et al. [43]. They randomized cervical SCI (C3-T1) levels of different grades of injury to early <72 hours surgery and late >5 days surgery. The study failed to show any difference in outcome between the two groups (mean follow up 305 days). Another aspect of the controversy surrounding surgical treatment timing is whether surgery, especially early surgery has any influence on complication rate or length of stay after SCI. It is obvious that surgery might be risky in more critically ill patients who sustain SCI; especially if the cervical injury is high, and is associated with other significant injuries. Both Vaccaro et al. [43] and Tator et al. [44] showed that there was no significant difference in the length of stay between operative and nonoperative groups or between the early versus late surgery groups. There is clear evidence [45] that injury severity is crucial in determining the ultimate outcome after traumatic SCI, but the potential impact of injury mechanism or SCI type remains elusive. Pollard and Apple [24] were unable to identify an association between mechanism of injury and neurological recovery or outcome. However, they were able to observe that patients with Brown-Sequard syndrome or a central cord syndrome had relatively improved outcomes. The relatively improved outcome in central cord syndrome patients when compared to other mechanisms of SCI is a notable observation and affects decision making regarding timing of surgery in this particular SCI model. Contrary to all other SCI types, central cord syndrome is one type where early surgery is not classically recommended in the acute setting; typically these patients are managed conservatively with a collar and rest. Recent consensus is that there is no standard recommendation regarding the role and timing of surgery in acute SCI [22]. There are guideline recommendations supporting the safety of early surgery (<72 hours) in hemodynamically stable patients, and data supports recommendations for urgent reduction of bilateral locked facets in patients with incomplete tetraplegia and urgent decompression in patients with neurological deterioration. There are also recommendations supporting decompression as reasonable for acute cervical SCI; when possible, excluding patients with life-threatening multisystem trauma, it is recommended that urgent decompression be performed within 24 hours of a SCI. There is class III evidence that early (<24 hours) surgery reduces length of stay in patients with acute SCI and may reduce postinjury medical complications [22, 23]. There is an obvious need for well-designed, executed, and randomized controlled trials comparing early versus late surgery in SCI. Questions regarding the relation between a particular SCI type, level or severity and surgery outcome would be logical. It has yet to be determined with certainty which type of SCI would benefit most, if at all, from early surgery.

Finally, it is worth mentioning that currently the Toronto group [23], in collaboration with Thomas Jefferson University and the Spine Trauma Study Group, has started a multicenter, prospective trial to evaluate the effect of early (<24 hours after injury) versus late (>24 hours) decompressive surgery for cervical SCI. The study (Surgical Timing in Acute Spinal Cord Injury Study, i.e., STASCIS) is not randomized because of the ethical concerns related to allocating a neurologically declining patient to a delayed decompression group. The study is currently open and when complete will have enrolled up to 450 patients.

## 4. Surgical Approach Choice

Spinal cord injury patients are a complex and heterogeneous group, as previously mentioned, therefore, a unified approach to the spine is impossible.

In acute SCI patients, the choice of the surgical procedure is a function of the objective of the surgery; fixation of instability and decompression of the spinal cord. The choice of one procedure or another is a function of the severity of injury, the level of injury, the mechanism of injury, and the location and the extent of compression. Goal setting before the procedure is critical to procedure choice; hence a surgical plan should be individualized to each patient. In patients with complete spinal cord injuries, overall outcome is generally poor, and usually the primary goal of surgery is spinal stabilization [46]. This allows the patient better postural management, reduces pain, improves pulmonary functions and allows the initiation of physical therapy and rehabilitation. Although spontaneous fusion is eventually the fate of many cases even without surgery, surgical stabilization allows the early benefit of fusion and helps prevent possible future kyphotic deformity.

In incomplete SCI patients, the choice of the procedure is dependent on the mechanism of injury and the other factors mentioned above. Approaches to the spine can be classified as anterior or ventral and posterior or dorsal. Either objective, that is, stabilization or decompression can be achieved from either route, but in severe cases combined anterior and posterior approaches (staged or in a single setting) may be required [47].

In acute traumatic SCI, the classic controversy of short versus long segment stabilization does not exist. Acute SCI trauma usually involves one or more specific segments and short segment instrumented fusion is therefore almost always the preferred choice. Posterior approaches are usually used in flexion type injuries and in most thoracolumbar injuries provided there is no severe ventral compression that is thought to be related to a partial SCI. Instrumentation can be lateral mass, Halifax clamp or wires in the cervical spine, transpedicular screws, and sublaminar hooks or wires in the thoracolumbar spine. Currently, the use of hooks or wires is very rare. A posterior approach allows easy and quick spinal decompression via laminectomy, if needed, and also allows ligamentotaxis in thoracolumbar injuries [48]. The ventral or anterior approach is used most commonly in cervical spine traumatic SCI, much less so in thoracolumbar injuries, because of the anatomical barrier of the chest and abdomen and the related difficulty in surgical approach. Anterior approaches are used in extension injuries and allow for corpectomy and ventral decompression if needed. Instrumentation can be anterior plates, cages, disc spacers, and anterior screws. An anterior approach can often be combined with a posterior approach; the sequence of this combination is an individual surgeon's choice. Several authors have attempted to devise an algorithm for decision making that employs the factors mentioned above, however approach choice remains individually tailored, by the surgeon, to meet the specific goals of the individual patient [49, 50]. From the stand point of evidence-based medicine, it is unlikely that evidence supporting one specific approach or another to treat a particular injury pattern, injury type, or level, is possible. The severity of injuries is too complex and heterogeneous to be broken down to essentially a handful types and there are multiple surgical options available and used by many surgeons.

## 5. Conclusion

Traumatic SCI is a major source of morbidity, mortality, and imposed cost on society as a whole. The failure of improvement in SCI outcomes despite extensive efforts is frustrating. Surgical treatment remains an essential tool in management and the controversy regarding the timing of surgery needs to be resolved. Collection of outcome data that would suffice as evidence-based is a huge undertaking, and hopefully this will be accomplished in the future. Presurgical reduction is considered safe and essential in the management of SCI with loss of alignment, at least as an initial step in the overall care of a SCI patient.

##  Conflict of Interests

The authors report no conflict of interests concerning the materials or methods used in this study or the findings specified in this paper.
